# C1QBP Modulates DNA Damage Response and Radiosensitivity in Hepatocellular Carcinoma by Regulating NF-κB Activity

**DOI:** 10.3390/ijms26104513

**Published:** 2025-05-09

**Authors:** Haitao Zhou, Yanjin Wu, Jiahui Meng, Xiaotong Zhao, Yujia Hou, Qin Wang, Yang Liu

**Affiliations:** State Key Laboratory of Advanced Medical Materials and Devices, Tianjin Key Laboratory of Radiation Medicine and Molecular Nuclear Medicine, Tianjin Institutes of Health Science, Institute of Radiation Medicine, Chinese Academy of Medical Sciences & Peking Union Medical College, Tianjin 300192, China; zhouhait20@163.com (H.Z.); wu1997814@163.com (Y.W.); mengjiahui@pumc.edu.cn (J.M.); zhaoxiaotong@irm-cams.ac.cn (X.Z.); s2023016008@pumc.edu.cn (Y.H.)

**Keywords:** C1QBP, NF-κB, HCC, DDR, radiosensitivity

## Abstract

C1QBP (Complement Component 1 Q Subcomponent-Binding Protein) plays a critical role in maintaining cellular metabolism, but its function in radiation-induced damage remains unclear. In this study, we generated C1QBP-deficient Huh-7 hepatocellular carcinoma (HCC) cells using CRISPR/Cas9 technology and observed that C1QBP deficiency significantly enhanced radiation-induced damage, as indicated by reduced cell proliferation, impaired colony formation, and increased γ-H2AX foci, a marker of DNA double-strand breaks. Additionally, C1QBP deficiency resulted in elevated phosphorylation of key DNA damage response (DDR) molecules, ATM and CHK2, and caused pronounced S phase cell cycle arrest. Mechanistic investigations revealed that C1QBP modulates NF-κB nuclear activity via the AMPK signaling pathway. The loss of C1QBP reduced NF-κB nuclear translocation, further exacerbating radiation-induced damage. Reintroducing C1QBP alleviated DNA damage, enhanced cell proliferation, and improved survival following radiation exposure. These findings highlight the critical role of C1QBP in modulating HCC cells radiosensitivity and underscore its potential as a therapeutic target to enhance radiotherapy outcomes.

## 1. Introduction

Hepatocellular carcinoma (HCC) is one of the most prevalent malignant tumors globally, with limited therapeutic options and high recurrence rates. Despite advancements in surgical resection, liver transplantation, and local therapies, radiotherapy (RT) remains a critical treatment modality for HCC [1]. However, radiotherapy often faces the challenge of radiation resistance, leading to suboptimal therapeutic outcomes. Increasing evidence suggests that the radiation sensitivity of HCC cells is influenced not only by their genetic characteristics but also by various intracellular regulatory mechanisms [2]. Radiation therapy primarily induces DNA damage, triggering the DNA damage response (DDR) to either repair the damage or initiate cell death pathways. Key molecules involved in DDR, including ATM, CHK2, and γ-H2AX, play crucial roles in determining cellular radiosensitivity. Abnormal activation of DDR pathways can reduce the efficiency of radiotherapy by enhancing DNA repair mechanisms, allowing cancer cells to survive and proliferate post-radiation exposure.

In the context of the immune microenvironment, the infiltration of suppressive immune cells and the abnormal expression of immune checkpoint molecules contribute to radiation resistance [2]. These seemingly independent regulatory axes may be functionally interconnected through specific hub molecules, among which Complement Component 1 Q Subcomponent-Binding Protein (C1QBP) has attracted significant attention due to its unique biological localization. C1QBP, also known as p32 or HABP1, is a highly conserved and multifunctional mitochondrial protein that forms a doughnut-shaped homotrimeric structure [3]. It is widely distributed across various subcellular compartments, with a predominant localization in mitochondria. Recent studies have revealed its critical role in maintaining oxidative phosphorylation (OXPHOS) and metabolic homeostasis by modulating mitochondrial protein translation [4]. The loss of C1QBP has been shown to significantly impair electron transport chain activity and reduce fatty acid β-oxidation efficiency, thereby affecting cellular energy metabolism and differentiation [5]. Beyond its metabolic functions, C1QBP is also involved in regulating mitochondrial dynamics. It upregulates the expression of mitochondrial fusion proteins MFN1 and MFN2, promotes autophagic degradation of Parkin to modulate mitochondrial morphology, and stabilizes the inner mitochondrial membrane by inhibiting OPA1 proteolytic processing [6]. As a multi-compartmental protein, C1QBP also participates in the regulation of immune and inflammatory responses, demonstrating strong signal integration capacity [5]. It acts as a high-affinity receptor for complement component C1q, blocking the activation of the classical complement pathway and exerting anti-inflammatory effects [7]. In summary, C1QBP is a multifunctional mitochondria-associated protein involved in regulating cellular energy metabolism, mitochondrial dynamics, immune and inflammatory responses, apoptosis, and pathogen infection, highlighting its significant physiological relevance and potential as a therapeutic target [3]. Recent studies have increasingly focused on its involvement in tumor radiation effects [7,8]. Studies have shown that C1QBP can enhance nuclease activity by interacting with MRE11, thereby promoting recombination repair [9]. Additionally, the loss of C1QBP leads to impaired T cell function, weakening the tumor’s immune response capability [10]. These findings highlight the significant role of C1QBP in cancer initiation and progression [11].

Studies have revealed the potential role of C1QBP in various cancers, including breast and lung cancer, where its expression is linked to tumor invasiveness and prognosis. High levels of C1QBP have been associated with poor patient prognosis [12,13]. It has been confirmed that high expression of C1QBP correlates with lower patient survival rates, and the loss of C1QBP suppresses HCC cell survival [12]. However, the impact of C1QBP loss on the radiation sensitivity of liver cancer cells and the underlying mechanisms remains unclear [14]. C1QBP is closely associated with cellular stress responses, and some studies have reported its loss enhances the chemotherapy sensitivity of triple-negative breast cancer (TNBC) cells by inhibiting NF-κB activation [15]. NF-κB is a key transcription factor that regulates essential biological processes such as cell survival, inflammation, and DNA damage repair [16,17].

Additionally, growing evidence highlights the significant role of the NF-κB signaling pathway in HCC pathogenesis, progression, and response to radiotherapy [18]. During radiation treatment, NF-κB activation is thought to promote tumor cell survival and repair, thereby potentially influencing radiation sensitivity [19]. However, it has not yet been reported whether C1QBP can modulate HCC radiation sensitivity through NF-κB modulation. Therefore, investigating the role of NF-κB in HCC radiation sensitivity, particularly in relation to its interaction with C1QBP, may provide new insights to enhance the efficacy of radiotherapy. In the NF-κB signaling pathway, NF-κB is not a single protein but a transcription factor complex composed of multiple subunits [20]. The most common and representative active form is a heterodimer consisting of p65 (also known as RelA) and p50, in which p65 plays a critical role in transcriptional activation [20]. The nuclear translocation of the p65 protein is a key hallmark of NF-κB pathway activation [21]. This subunit is responsible for regulating various biological processes, including inflammatory responses, apoptosis, and immune responses [21]. Collectively, this study aims to fill this gap by exploring the role of C1QBP in regulating radiation sensitivity in HCC cells, with a particular focus on its modulation of the NF-κB signaling pathway. By elucidating its regulatory mechanisms in key signaling pathways, such as AMPK and NF-κB, our findings provide valuable insights into the molecular basis of radioresistance in HCC cells. Furthermore, this research highlights the potential of targeting C1QBP as a novel strategy to improve the efficacy of radiotherapy for HCC patients.

## 2. Results

### 2.1. C1QBP KO Increases the Radiosensitivity of HCC Cells

**Figure 1:**
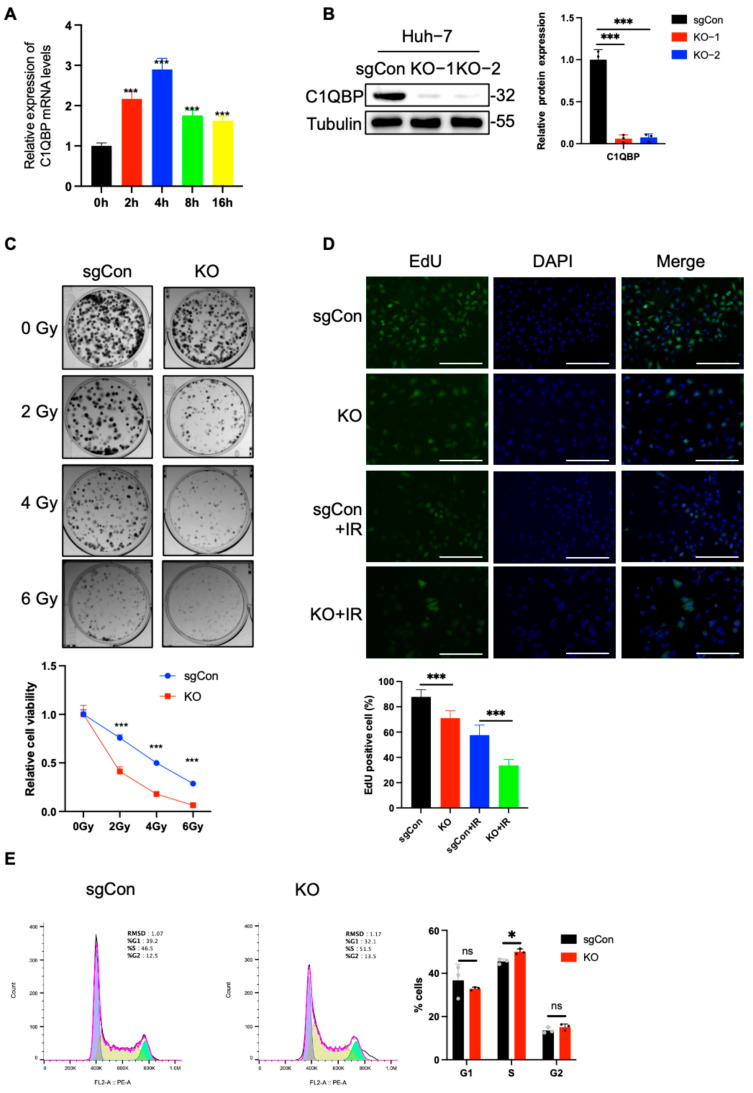
C1QBP KO increases the radiosensitivity of HCC cells. (**A**) qPCR analysis showed the expression of C1QBP in Huh-7 cells at different time points after exposure to 4 Gy of radiation. (**B**) Western blot confirming the successful knockout of C1QBP in Huh-7 cells via CRISPR/Cas9. C1QBP were normalized to Tubulin as a loading control, and the resulting data were used to generate quantitative statistical graphs. (**C**) Colony formation assay assessing the proliferation of sgCon and C1QBP-deficient Huh-7 cells after 0, 2, 4, or 6 Gy radiation exposure. (**D**) EdU incorporation assay measuring the proliferative capacity of sgCon and C1QBP-deficient Huh-7 cells after 6 Gy radiation. Scale bars, 100 µm. (**E**) PI staining showing the cell cycle distribution of sgCon and C1QBP-deficient Huh-7 cells. * *p* < 0.05, *** *p* < 0.001 by two-tailed unpaired Student’s *t*-test or two-way ANOVA.

C1QBP plays an important role in cell metabolism, but its role in radiation-induced damage has not been studied [11,22]. Results of qPCR analysis in our current study showed that C1QBP expression increased in Huh-7 cells at various time points after radiation exposure (Figure 1A), suggesting its involvement in the DDR of HCC. To investigate the role of C1QBP in the DDR to radiation in HCC, we used the CRISPR/Cas9 system to knock out the C1QBP gene in Huh-7 cells. Western blot confirmed a significant reduction in C1QBP protein expression (Figure 1B), indicating the successful generation of C1QBP-deficient Huh-7 cells. We first assessed the impact of C1QBP on Huh-7 cell proliferation after irradiation. Colony formation assays showed reduction in colony number following radiation exposure in sgCon cells (Figure 1C). Notably, C1QBP-deficient cells exhibited even fewer colonies than irradiated sgCon cells, indicating that the loss of C1QBP exacerbates radiation-induced damage. Interestingly, C1QBP knockout also resulted in a decrease in colony formation even in the absence of radiation, underscoring its importance for normal cell growth and viability.

Next, we examined cell proliferation activity using EdU staining. The EdU incorporation assay revealed that C1QBP-deficient cells showed a significantly lower percentage of EdU-positive cells after radiation exposure compared to irradiated sgCon cells (Figure 1D). This indicates that C1QBP knockout dramatically impairs the proliferative capacity of HCC cells post-irradiation. Similarly, in the absence of radiation, C1QBP knockout reduced the proportion of EdU-positive cells, further confirming its role in regulating cell proliferation under normal conditions. To explore the impact of C1QBP deficiency on cellular homeostasis, we assessed changes in the cell cycle using PI staining. We observed significant cell cycle arrest at the S phase in C1QBP-deficient cells (Figure 1E), suggesting that the loss of C1QBP exacerbates cell cycle disruption. Together, these findings demonstrate that C1QBP deficiency significantly increases the radiosensitivity of Huh-7 HCC cells.

### 2.2. C1QBP KO Aggravates Radiation-Induced DNA Damage

**Figure 2:**
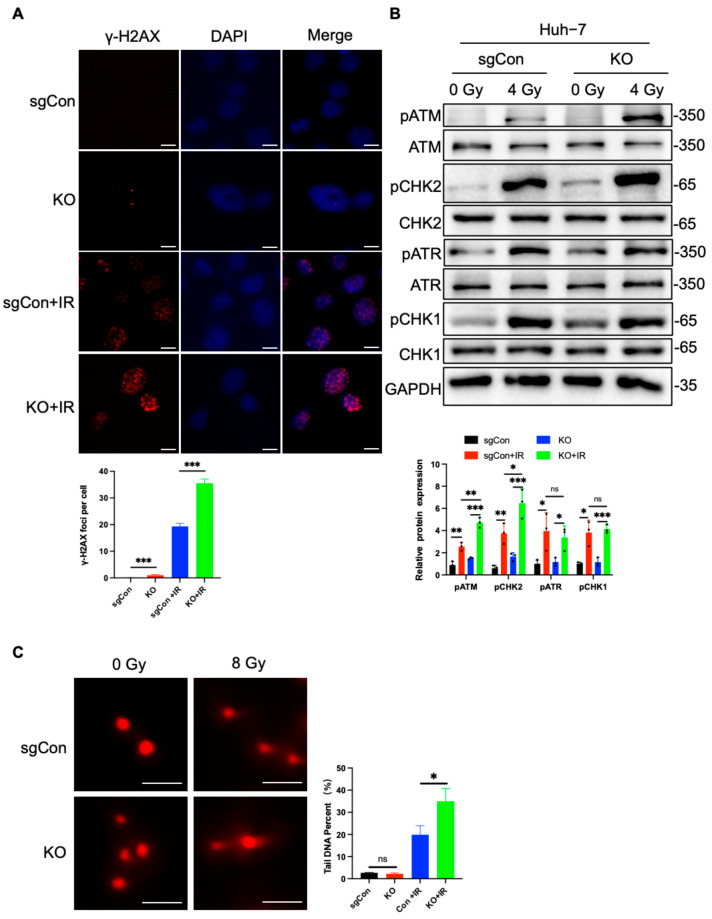
C1QBP deficiency exacerbates radiation-induced DNA damage in Huh-7 liver cancer cells. (**A**) γ-H2AX foci formation analysis in sgCon and C1QBP-deficient Huh-7 cells following 6 Gy radiation exposure. Scale bars, 10 µm. (**B**) Western blot analysis of DNA damage response proteins, including phosphorylated ATM and CHK2, in sgCon and C1QBP-deficient Huh-7 cells after radiation. All band intensities were quantified using GAPDH as a loading control. (**C**) Comet assay measuring DNA strand breaks in sgCon and C1QBP-deficient Huh-7 cells following 8 Gy radiation exposure. Scale bars, 20 µm. * *p* < 0.05, ** *p* < 0.01, and *** *p* < 0.001 by two-tailed unpaired Student’s *t*-test or two-way ANOVA.

Given that C1QBP deficiency increases cellular radiation sensitivity, we next sought to investigate whether C1QBP deficiency exerts its effect by aggravating radiation-induced DNA damage. γ-H2AX, the phosphorylated form of histone H2AX at Ser139, serves as a sensitive and early biomarker of DNA double-strand breaks [23]. It plays a critical role in recruiting DNA repair factors to the site of damage and is widely used to quantify DNA damage in response to radiation [23,24]. In C1QBP knockout Huh-7 cells, we detected a significant increase in γ-H2AX focus formation after radiation exposure using immunofluorescence (Figure 2A), indicating a higher level of DNA double-strand breaks (DSBs). Western blot analysis further confirmed that the expression of radiation-induced DNA damage-related genes was significantly upregulated in C1QBP-deficient cells (Figure 2B). Specifically, proteins associated with DNA repair and cell cycle checkpoints, such as phosphorylation of ATM (Ataxia-Telangiectasia Mutated) and CHK2 (checkpoint kinase 2), were expressed at higher levels in C1QBP knockout cells (Figure 2B), indicating that the absence of C1QBP leads to an overactivation of the cellular response to radiation. In addition, ATR (ATM and Rad3-related) and Chk1 (checkpoint kinase 1) are key factors in the cellular response to DNA damage and replication stress [25]. Their expression and activation are significantly altered following exposure to ionizing radiation [26]. However, in this study, the loss of C1QBP did not lead to an increase in pATR and pChk1, but instead resulted in an increase in pATM and pChk2 (Figure 2B).

Additionally, comet assay results showed a significant increase in tail DNA percentage in C1QBP-deficient Huh-7 HCC cells following radiation exposure (Figure 2C). Tail DNA percentage, defined as the proportion of total DNA present in the comet tail, was used as a quantitative indicator of DNA strand breaks [27]. The increased tail DNA percentage typically reflects DNA strand breaks and impaired repair capacity, further validating the severity of DNA damage in these cells [28]. This result is consistent with our observed reduction in cell proliferation and clonogenic potential post-radiation.

Taken together, these findings suggest that C1QBP plays a crucial role in mitigating radiation-induced DNA damage. The absence of C1QBP leads to an exaggerated cellular response to radiation, resulting in enhanced DNA damage accumulation.

### 2.3. C1QBP Regulates NF-κB Activity Via AMPK

**Figure 3:**
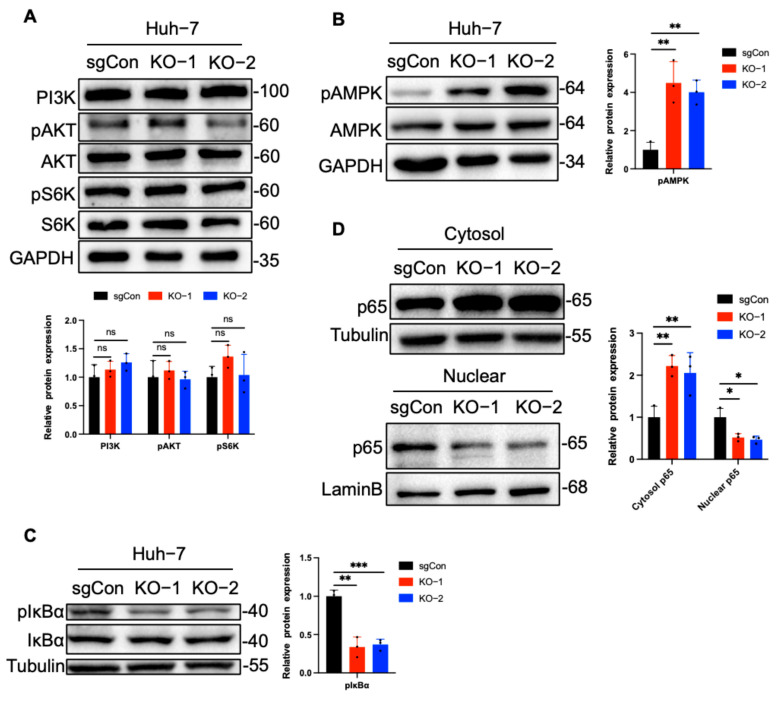
C1QBP modulates the AMPK-NF-κB pathway in response to radiation. (**A**) Western blot analysis of phosphorylation and basal expression levels of PI3K, Akt, and S6K in sgCon and C1QBP-deficient cells. (**B**) Western blot analysis of p-AMPK levels in sgCon and C1QBP-deficient Huh-7 cells following radiation exposure. (**C**) Phosphorylation status of IκBα assessed via Western blot in sgCon and C1QBP-deficient cells. (**D**) Nuclear–cytoplasmic fractionation analysis showing NF-κB nuclear translocation in sgCon and C1QBP-deficient Huh-7 cells. Cytoplasmic p65 was quantified relative to Tubulin, and nuclear p65 was quantified relative to Lamin B. The normalized values were used to generate quantitative statistical graphs. * *p* < 0.05, ** *p* < 0.01, and *** *p* < 0.001 are considered statistically significant differences.

To further investigate how C1QBP affects cellular radiosensitivity, we analyzed several classical signaling pathways, particularly the AMPK pathway and the PI3K-Akt-S6K pathway. AMPK plays a crucial role in regulating radiosensitivity. Activation of AMPK can enhance radiotherapy efficacy by inhibiting the mTOR pathway, inducing G2/M arrest, interfering with DNA repair, and amplifying stress responses, ultimately promoting apoptosis [29,30]. AMPK activators such as AICAR and metformin have been shown to increase radiosensitivity in various cancer models [31,32]. Conversely, the PI3K–Akt–S6K pathway supports cell survival and DNA repair following ionizing radiation [33,34]. Its inhibition impairs DNA damage repair and promotes apoptosis [35]. Therefore, we investigated whether C1QBP deficiency sensitizes HCC cells to radiation by suppressing the PI3K–Akt–S6K pathway. The levels of PI3K and the phosphorylation levels of Akt and S6K were detected by Western blot, and no significant changes were observed in either their phosphorylation status or basal expression (Figure 3A). Notably, a marked increase in the phosphorylation of AMPK in C1QBP knockout cells (Figure 3B). As a key sensor of cellular energy status, AMPK is activated under metabolic stress conditions. Studies have shown that AMPK can inhibit the phosphorylation and degradation of IκBα, thereby stabilizing its expression and maintaining the inhibitory effect of IκBα on NF-κB signaling [36]. Consequently, we examined the phosphorylation status of IκBα and the phosphorylated expression of IκBα was significantly decreased (Figure 3C), correlating with the elevated phosphorylation of AMPK. Since NF-κB functions mainly as a transcription factor in the nucleus, we performed nuclear-cytoplasmic fractionation to assess its nuclear translocation. Western blot analysis showed that the nuclear level of NF-κB was significantly reduced in C1QBP-knockout Huh-7 cells, while the cytoplasmic level of p65 was markedly increased (Figure 3D), indicating that loss of C1QBP may inhibit the nuclear translocation of NF-κB. This finding further substantiates the potential role of C1QBP in regulating NF-κB signaling, particularly in the context of radiation sensitivity.

### 2.4. C1QBP Affects Radiation-Induced DNA Damage by Regulating NF-κΒ Activity

**Figure 4:**
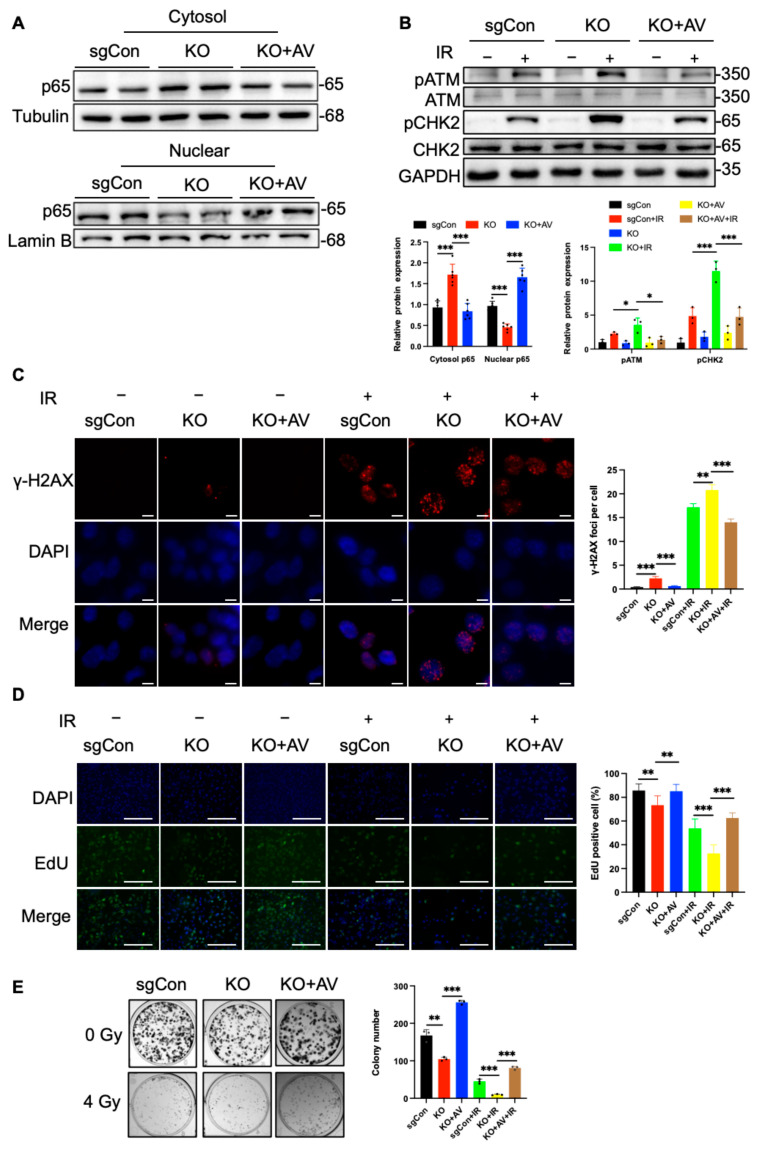
The activation of NF-κB alleviates radiation-induced DNA damage and cell proliferation in HCC cells. (**A**) Western blot detection of p65 protein changes in C1QBP KO cells treated with NF-κB activator and sgCon, and C1QBP KO cells treated with DMSO, respectively. Cytoplasmic p65 was quantified relative to Tubulin, and nuclear p65 was quantified relative to Lamin B. The normalized values were used to generate quantitative statistical graphs. (**B**) Western blot analysis of DDR pathway proteins (p-ATM and p-CHK2) in response to NF-κB activation following 4 Gy radiation exposure. (**C**) γ-H2AX immunofluorescence staining showing DNA double-strand break formation in C1QBP KO cells treated with NF-κB activator or sgCon, C1QBP KO cells treated with DMSO after 6 Gy radiation. Scale bars, 10 µm. (**D**) EdU incorporation assays evaluating cell proliferation in C1QBP KO cells treated with NF-κB activator or sgCon, and C1QBP KO cells treated with DMSO. Scale bars, 100 µm. (**E**) Colony formation assays assessing clonogenic potential in response to NF-κB activation following 4 Gy radiation exposure. * *p* < 0.05, ** *p* < 0.01, and *** *p* < 0.001 are considered statistically significant differences.

**Figure 5:**
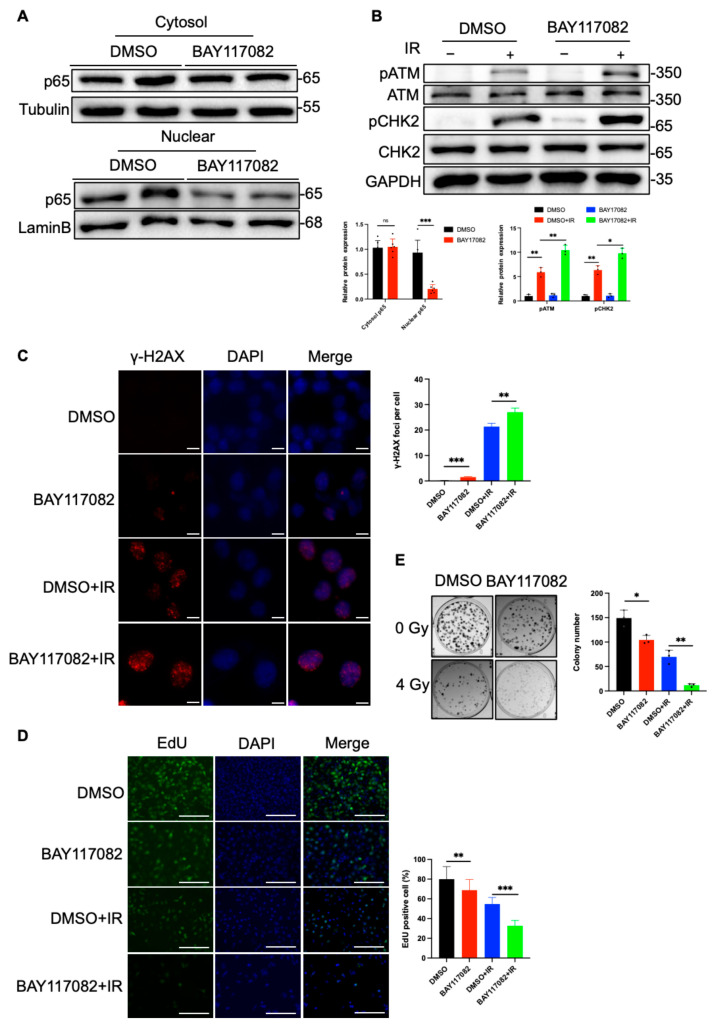
Inhibition of NF-κB increases radiation-induced DNA damage and cell proliferation in HCC cells. (**A**) Western blot detection of p65 protein changes in WT treated with NF-κB activator or DMSO, respectively. (**B**) Western blot analysis of DDR pathway proteins (p-ATM and p-CHK2) in response to pharmacological inhibition following 4 Gy radiation exposure. (**C**) γ-H2AX immunofluorescence staining showing DNA double-strand break formation in WT treated with NF-κB activator or DMSO after 6 Gy radiation. Scale bars, 10 µm. (**D**) EdU incorporation assays evaluating cell proliferation in WT treated with BAY17082 or DMSO. Scale bars, 100 µm. (**E**) Colony formation assays assessing clonogenic potential in response to pharmacological inhibition following 4 Gy radiation exposure. * *p* < 0.05, ** *p* < 0.01, and *** *p* < 0.001 are considered statistically significant differences.

We next investigated the role of NF-κB in the radiation-induced damage response and evaluated the impact of its inhibition on the radiosensitivity of liver cancer cells. To determine whether C1QBP deficiency exacerbates radiosensitization by inhibiting NF-κB, we treated C1QBP knockout Huh-7 cells with NF-κB activator 1 (AV) and wild-type (WT) Huh-7 cells with BAY117082, an NF-κB inhibitor. The results showed that BAY117082 reduced the expression of NF-κB subunit p65 in the nucleus, while NF-κB agonists increased the protein expression of p65. As a key subunit of NF-κB, the changes in p65 also proved that the two agents could inhibit or increase NF-κB (Figure 4A and Figure 5A). Western blotting analysis showed that after 4 Gy radiation exposure, the phosphorylation levels of key molecules in the DDR pathway, the levels of p-ATM and p-CHK2 were reduced in C1QBP knockout Huh-7 cells treated with the NF-κB activator (AV) (Figure 4B). Notably, p-ATM and p-CHK2 were significantly elevated after treated with the NF-κB inhibitor of BAY117082 (Figure 5B). This suggests that the reduction in NF-κB signaling due to C1QBP knockout exacerbates radiation-induced damage.

The results of γ-H2AX immunofluorescence staining showed that in C1QBP knockout cells treated with AV, the formation of γ-H2AX foci after radiation treatment was significantly reduced compared with the C1QBP-KO group (Figure 4C). Conversely, the number of γ-H2AX foci increased in WT cells treated with BAY117082 after radiation treatment (Figure 5C). These results further support the notion that NF-κB inhibition enhances the DNA damage response induced by radiation, thereby increasing the radiosensitivity of liver cancer cells.

To further assess the effect of NF-κB inhibition on cell proliferation, we conducted EdU incorporation assays. The results showed that the percentage of EdU-positive cells was restored in C1QBP-deficient cells treated with NF-κB activator post radiation (Figure 4D), whereas NF-κB inhibition in WT cells treated with BAY117082 showed a significant reduction in the percentage of EdU-positive cells post radiation (Figure 5D). These findings suggest that NF-κB inhibition significantly impairs the proliferative capacity of Huh-7 cells after radiation, further supporting the role of NF-κB in regulating cell proliferation. Consistent with these results, colony formation assays demonstrated that the number of colonies in irradiated Huh-7 cells increased upon NF-κB activation (Figure 4E), while a marked decrease in colony formation was observed in cells treated with BAY117082 post radiation (Figure 5E). This phenomenon suggests that NF-κB inhibition not only suppresses cell proliferation but also potentially compromises DNA repair, thereby reducing the ability of cells to form colonies.

### 2.5. Reintroduction of C1QBP Ameliorates Radiation-Induced DNA Damage

**Figure 6:**
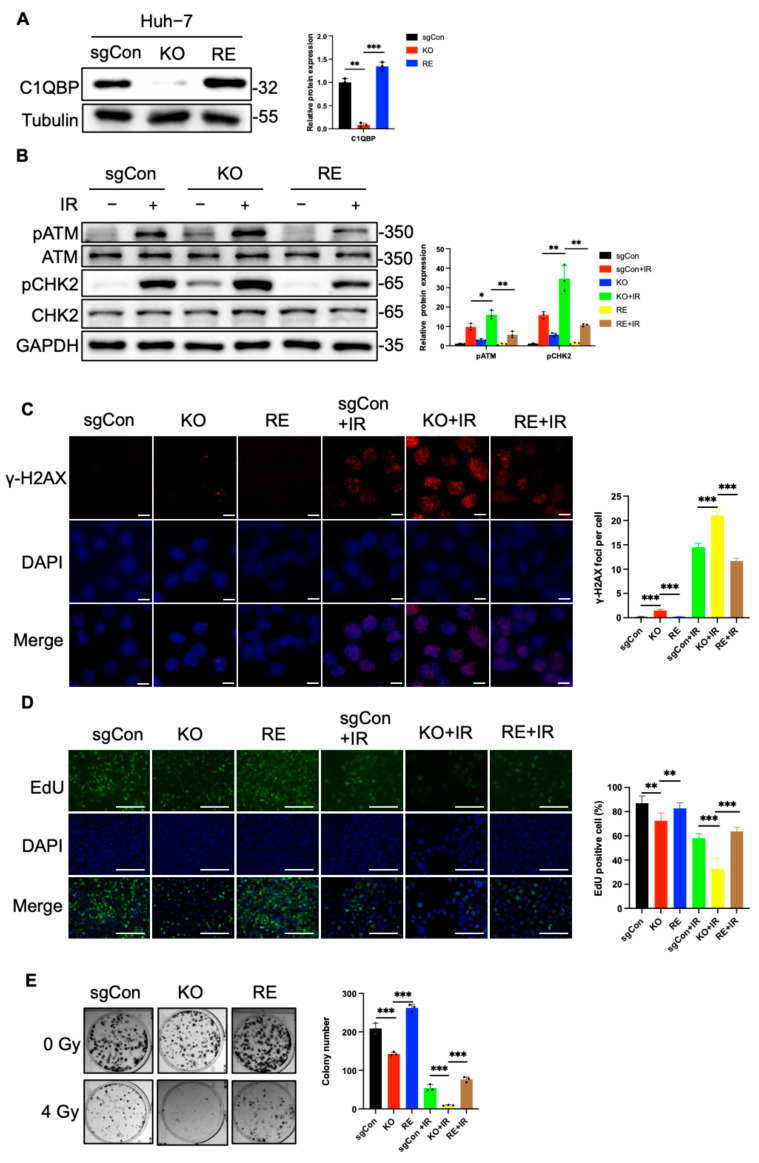
RE of C1QBP alleviates radiation-induced DNA damage and enhances cell proliferation in Huh-7 cells. (**A**) Western blot confirming successful RE of C1QBP in Huh-7 cells. (**B**) Western blot analysis of radiation-responsive proteins p-ATM and p-CHK2 in C1QBP-reintroduced, C1QBP-knockout, and sgCon Huh-7 cells following 4 Gy radiation exposure. (**C**) Immunofluorescence analysis of γ-H2AX foci formation in C1QBP-reintroduced, C1QBP-knockout, and sgCon Huh-7 cells after 6 Gy irradiation. Scale bars, 10 µm. (**D**) EdU incorporation assay evaluating cell proliferation in C1QBP-reintroduced, C1QBP-knockout, and sgCon Huh-7 cells after 6 Gy radiation. Scale bars, 100 µm. (**E**) Colony formation assay assessing survival and proliferation of C1QBP-reintroduced, C1QBP-knockout, and sgCon Huh-7 cells following 4 Gy radiation exposure. * *p* < 0.05, ** *p* < 0.01, and *** *p* < 0.001 are considered statistically significant differences.

To further investigate the impact of C1QBP on radiation sensitivity, we constructed a plasmid for the reintroduction (RE) of C1QBP into Huh-7 cells. Successful RE of C1QBP was confirmed by Western blot (Figure 6A). Upon exposure to 4 Gy of radiation, Huh-7 cells with C1QBP reintroduction exhibited significantly reduced activation of the radiation-responsive proteins p-ATM and p-CHK2 compared with C1QBP-deficient cells, indicating that increased C1QBP expression confers radiation resistance (Figure 6B). Immunofluorescence analysis revealed that the number of rH2AX foci in C1QBP-reintroducing Huh-7 cells was markedly reduced compared to cells with C1QBP knockout after 6 Gy irradiation (Figure 6C). Notably, the number of rH2AX foci in C1QBP- reintroducing cells was even lower than that in sgCon cells after 6 Gy irradiation (Figure 6C). This suggests that reintroduction of C1QBP effectively alleviates radiation-induced DNA damage.

Additionally, results from EdU staining demonstrated that after 6 Gy radiation, a higher proportion of EdU-positive cells were observed in the C1QBP-reintroducing group compared to both the C1QBP-knockout and sgCon groups (Figure 6D), indicating that C1QBP expression significantly alleviated radiation-induced inhibition of cell proliferation. Consistently, colony formation assays revealed that C1QBP- reintroducing Huh-7 cells formed more colonies than both the C1QBP-knockout and sgCon groups following 4 Gy radiation exposure (Figure 6E), further supporting that C1QBP promotes cell survival and proliferation after irradiation.

Taken together, these findings demonstrate that reintroducing C1QBP substantially enhances the proliferative capacity of Huh-7 cells under radiation conditions while mitigating radiation-induced DNA damage. These results provide a theoretical foundation for C1QBP as a potential target to enhance the efficacy of radiation therapy in liver cancer.

## 3. Materials and Methods

### 3.1. Cell Culture

The human liver cancer cell line Huh-7 was obtained from American Type Culture Collection (ATCC, Manassas, VA, USA). C1QBP knockout cells were generated using CRISPR/Cas9 technology. Cells transduced with lentivirus expressing a non-specific sgRNA were used as control (sgCon), and C1QBP knockout cells were infected with lentivirus encoding sgRNA targeting C1QBP. Viral supernatant was added to the culture medium of Huh-7 cells for infection. After 48 h, the cells were subjected to selection with 2 μg/mL puromycin to establish stable cell lines. The efficiency of gene knockout was subsequently verified by Western blot analysis. All the cells were cultured in the humidified incubator with 5% CO_2_ at the temperature of 37 °C. The complete medium consisted of 90% DMEM medium (Gibco, Carlsbad, CA, USA), 10% fetal bovine serum (Gibco, Carlsbad, CA, USA), 100 ug/mL streptomycin (Gibco, Carlsbad, CA, USA), and 100 U/mL penicillin (Gibco, Carlsbad, CA, USA).

### 3.2. Colony Formation

C1QBP KO and sgCon cells were seeded in 6-well plates at a density of 2000 cells per well. After 24 h, cells were irradiated with 0, 2, 4, or 6 Gy. After irradiation, cells were incubated for 6 days to form countable colonies. Colonies were fixed with 4% buffered paraformaldehyde and stained with 0.25% crystal violet. The colony count was determined using ImageJ software (OpenComet v1.3.1). 

### 3.3. Western Blot

Cells were washed in phosphate-buffered saline (PBS) and lysed in RIPA buffer containing proteinase inhibitor (Yeason, Shanghai, China) and phosphatase inhibitor cocktail (Yeason, Shanghai, China) for 10 min. The lysates were harvested by centrifugation at 12,000 rpm for 15 min at 4 °C. The samples were then denatured at 95 °C for 10 min, and the protein concentration was quantified by BCA assay kits (Beyotime, Shanghai, China). Proteins samples were separated on 8% or 10% acrylamide SDS-PAGE gels and then transferred onto PVDF membranes (MF-Millipore). PVDF membranes were blocked using 3% BSA (Table 1) in TBS containing 0.1% Tween-20 (TBST) for 1 h. Then, membranes were incubated with primary antibodies (Table 2) at 4 °C overnight, followed by incubation with secondary antibodies in TBST for 1 h at room temperature.

### 3.4. q-PCR

The total RNA was extracted by TRIzol and reversed-transcribed using a Takara qPCR kit. q-PCR was performed with SYBR green PCR master mix and ran on an RT-qPCR system (Bio-Rad CFX Connect Real-Time PCR Detection System). The data were analyzed using the 2−Δ∆Ct method normalized to Actin.

### 3.5. Cell Cycle Arrest Analysis

Cells were dissociated with 10% FBS at 8 h post IR, and then centrifuged at 2500 rpm for 5 min at 4 °C. The pellets were washed with PBS and fixed with 75% ethanol at 4 °C for 2 h. Fixed cells were washed with PBS and resuspended in propidium iodide (PI) with DNase and sodium citrate at 37 °C. After incubation for 10 min, the cells were washed with PBS and analyzed using a flow cytometer. The cell cycle was analyzed using FlowJo software.

### 3.6. EdU Staining

C1QBP knockout and sgCon Huh-7 cells were incubated with 10 µM EdU for 2 h after 6 Gy irradiation or without irradiation. EdU labeling was performed using the EdU assay kit (Ribobio, guangzhou, China), and EdU incorporation into cells was detected using a confocal microscope. The percentage of EdU-positive cells was calculated by dividing the number of EdU-labeled cells by the total number of DAPI-stained nuclei in each field.

### 3.7. Immunofluorescence Staining

C1QBP knockout and sgCon Huh-7 cells were seeded in confocal dishes. After 24 h, they were irradiated with 6 Gy or left untreated. Then, 6 h post-irradiation, the cells were fixed at room temperature in 4% paraformaldehyde for 30 min. Then, they were washed three times with PBS containing 0.3% Tween 20. The cells were permeabilized using 0.3% Triton X-100 for 10 min, followed by three washes with PBS containing 0.1% Tween 20. Subsequently, the cells were blocked with 5% BSA in PBS at room temperature for 2 h and then incubated in a γ-H2AX antibody (1:500 in 5% BSA) overnight at 4 °C.

The primary antibody was washed three times, and then the cells were incubated with the secondary antibody at room temperature for 1 h. The coverslips were mounted onto the cells using DAPI mounting medium. All images were acquired using a confocal microscope.

### 3.8. Comet Assay

Cells were seeded and cultured for 48 h, followed by 8 Gy irradiation or no irradiation. Then, cells were collected and resuspended in PBS. The comet assay was performed according to the OxiSelectTM Comet Assay Kits (Cell Biolabs, San Diego, CA, USA, Cat# STA-351). Comet assay images were analyzed using OpenComet, a plugin for ImageJ (OpenComet v1.3.1). The tail DNA percentage was quantified for each cell using OpenComet, and statistical analyses were performed to assess differences between experimental groups.

### 3.9. Plasmid Transfection

Cells were plated in 6-well plates and allowed to reach 70–80% confluence before transfection. Lipofectamine 2000 (Invitrogen, Carlsbad, CA, USA) was used to mediate transfection, following the manufacturer’s protocol. For each well, 2 μg of plasmid DNA was diluted in Opti-MEM and mixed with the appropriate volume of Lipofectamine 2000. The mixture was gently blended and incubated at room temperature for 15 min to allow complex formation. The resulting transfection complexes were then added dropwise to the cells. After 24 h of incubation, cells were harvested for downstream analyses.

### 3.10. CRISPR/Cas9

The target gene was first identified through the NCBI database. Subsequently, the CRISPick online tool (https://portals.broadinstitute.org/gppx/crispick/public) developed by the Broad Institute was utilized to design sgRNAs. URL (accessed on 11 October 2024). Exons 1 and 2 of C1QBP were selected as target regions for guide RNA design.

HEK-293T cells were seeded in 15 cm dishes and cultured until they reached approximately 70% confluence. Prior to transfection, the cells were incubated in serum-free DMEM for 1 h. To generate knockout viruses, cells were transfected with 15 μg of the LentiCRISPRv2 plasmid harboring either sgRNA targeting the desired gene or a control sequence (details provided in Table 3), along with 3.75 μg of pVSVg, 11.25 μg of psPAX8, and 90 μL of PEI. Following transfection, cells were incubated in serum-free DMEM for 6 to 8 h before replacing the medium with 20 mL of DMEM supplemented with 10% FBS. After 24 h, the supernatant was collected and the medium was refreshed with another 20 mL of 10% FBS DMEM for an additional 24-h culture. The collected viral supernatants were pooled and centrifuged at 1000× *g* for 5 min at 4 °C. Finally, the virus-containing supernatant was filtered through 0.45 μm nylon membrane filters to complete the purification process.

### 3.11. Statistical Analysis

All statistical analyses were performed using GraphPad Prism 9.0. Results are expressed as mean ± standard error of the mean (SEM). Comparisons between groups were analyzed using a two-tailed unpaired Student’s *t*-test or a two-way analysis of variance (ANOVA). Differences were considered statistically significant when *p* < 0.05 (* *p* < 0.05, ** *p* < 0.01, *** *p* < 0.001).

## 4. Discussion

Hepatocellular carcinoma continues to pose a significant clinical challenge because of its intrinsic resistance to radiotherapy. This resistance arises from complex interactions among the DNA damage response (DDR), various cellular signaling pathways, and the tumor microenvironment. Our study identifies C1QBP as a novel modulator in HCC cells radioresistance and elucidates its potential role in regulating DDR and NF-κB signaling. These findings provide new insights into the molecular basis of radioresistance in HCC cells and suggest C1QBP as a promising therapeutic target for enhancing the effectiveness of radiotherapy.

Our results demonstrate that C1QBP is significantly upregulated in HCC tissues and radioresistant HCC cells, consistent with previous reports that link C1QBP to tumor progression and therapy resistance in various cancers. Functional assays revealed that the loss of C1QBP significantly increased the radiosensitivity of Huh-7 HCC cells. CRISPR/Cas9-mediated knockout of C1QBP resulted in marked suppression of proliferation, as evidenced by reduced colony formation and EdU staining post-irradiation. Our data indicate that the heightened sensitivity is directly associated with increased DNA damage, indicated by the substantial formation of γ-H2AX foci, a hallmark of radiation-induced DSBs.

Previous studies have implicated C1QBP in diverse cellular processes, including metabolism, cell cycle regulation, and stress responses, yet its role in radiation damage have not been fully elucidated [9,37]. Here, we demonstrated that DDR signaling was significantly amplified in C1QBP-deficient cells after radiation exposure. Western blot analyses revealed increased activation of key DDR proteins, including phosphorylated ATM and CHK2, suggesting that the absence of C1QBP triggers excessive DDR signaling. This heightened response may overwhelm DNA repair capacity, resulting in persistent DNA damage and cellular dysfunction. Consistent with these findings, comet assays, which demonstrated significantly increased DNA fragmentation in C1QBP-deficient cells, further validate the impaired repair and sustained DNA damage. Collectively, these results provide insights into the link between C1QBP and radiosensitivity, expanding our understanding of its role in radiation-induced cellular responses.

Our mechanistic investigations revealed that C1QBP regulates radiation responses through the NF-κB pathway. NF-κB is a key transcription factor involved in controlling immune responses, inflammation, and cell survival [38,39]. Studies have demonstrated the involvement of NF-κB in regulating cell survival and repair mechanisms in response to radiation [22,23]. However, its connection with C1QBP had not been previously reported. In this study, C1QBP deficiency led to significantly reduced NF-κB nuclear translocation, suggesting that C1QBP facilitates NF-κB activation in response to radiation. Western blot analysis further demonstrated that the increased radiosensitivity associated with C1QBP deficiency is closely linked to the suppression of the NF-κB signaling pathway. Experiments using pharmacological activators and inhibitors of NF-κB reinforced the role of this pathway in modulating DDR and cellular survival post-irradiation. Inhibition of NF-κB activity exacerbated DNA damage and reduced proliferation, while its activation partially rescued these effects. The exact molecular interactions between C1QBP and NF-κB warrant further investigation, but our findings suggest that C1QBP may function as a mediator linking DDR and NF-κB signaling in the context of radioresistance.

To validate the protective role of C1QBP, we reintroduced C1QBP into knockout Huh-7 cells. Remarkably, the reintroduction of C1QBP significantly reduced γ-H2AX foci formation and restored cell proliferation after radiation, as evidenced by improved results in EdU staining and colony formation assays. These observations confirm that C1QBP mitigates radiation-induced DNA damage and supports cellular recovery, emphasizing its potential as a therapeutic target for enhancing radiotherapy outcomes.

In conclusion, this study demonstrates that C1QBP plays a pivotal role in the radiation response of HCC cells. The absence of C1QBP increases radiosensitivity and amplifies radiation-induced DNA damage, underscoring its crucial function in promoting radioresistance. By modulating the NF-κB pathway, C1QBP contributes to protecting HCC cells from radiation damage. Moreover, the reintroduction of C1QBP mitigates radiation-induced damage, emphasizing its therapeutic potential in enhancing the efficacy of radiotherapy for HCC cells. Future studies should investigate the role of C1QBP in other cancer types and examine its interactions with additional DDR pathways. Such research could provide further insights into the broader implications of C1QBP in radiation responses and its viability as a universal target in cancer therapy.

## Figures and Tables

**Table 1:** Main reagents.

Reagent	Source	Identifer
BAY117082	MCE	Cat# HY-13453
NF-κB activator 1	MCE	Cat# HY-134476
BSA-V	Solarbio	Cat#A8020
Tween 20	Solomen	Cat#T8220
Triton X-100	Solarbio	Cat#T8200

**Table 2:** Antibodies for Western blot.

Antibody	Source	Identifer
Anti-C1QBP	Cell Signaling	Cat# 6502
Anti-Tubulin	Proteintech	Cat# 11224
Anti-p-ATM	Cell Signaling	Cat# 5883
Anti-ATM	Cell Signaling	Cat# 2873
Anti-p-CHK2	Cell Signaling	Cat# 2197
Anti-CHK2	Cell Signaling	Cat# 2662
Anti-p-ATR	Cell Signaling	Cat# 30632
Anti-ATR	Cell Signaling	Cat# 2790
Anti-p-CHK1	Cell Signaling	Cat# 12302
Anti-CHK1	Cell Signaling	Cat# 2360
Anti-p-AMPK	Cell Signaling	Cat# 50081
Anti-AMPK	Cell Signaling	Cat# 5831
Anti-p65	Cell Signaling	Cat# 8242
Anti-GAPDH	Proteintech	Cat# 60004
Anti-γH2AX	Sigma	Cat# 05636
Anti-FLAG	Proteintech	Cat# 20543

**Table 3:** sgRNA sequences for CRISPR/Cas9.

Gene	Forward	Reverse
*C1QBP KO1*	GCGTGCGCGCAGGTTCCGAG	CTCGGAACCTGCGCGCACGC
*C1QBP KO2*	ACGGAGGAGCCCAGCACACG	CGTGTGCTGGGCTCCTCCGT

## Data Availability

All data are available within the text.

## Abbreviations

DDR: DNA damage response; ATM: Ataxia-Telangiectasia Mutated; CHK2: checkpoint kinase 2; DSBs: DNA double-strand breaks; HCC: hepatocellular carcinoma; C1QBP: Complement Component 1 Q Subcomponent-Binding Protein; PBS: phosphate-buffered saline; RE: reintroduction; RT: radiotherapy; and AV: NF-κΒ activator 1.
